# Effect of feeding genetically modified Bt-corn on allergic disease

**DOI:** 10.1186/2045-7022-1-S1-P13

**Published:** 2011-08-12

**Authors:** Daniela Reiner, Rui-Yun Lee, Michelle M Epstein

**Affiliations:** 1 Medical University of Vienna, Vienna, Austria

## 

The rising prevalence of allergic disease in the last decades is unexplained. However, it has been postulated that the widespread introduction of genetically modified (GM) foods since 1996 may play a role in this evolving allergic disease epidemic. Currently, the most common GM plant is the genetically engineered Bacillus thuringiensis (Bt)-corn. This transgene confers resistance against corn borers leading to an enormous economic benefit. Corn products are found in a diverse variety of foodstuffs. Our hypothesis is that Bt-corn consumption influences allergic disease. We sought to determine whether feeding Bt-corn to mice would influence allergen-induced disease to a non-crossreactive allergen. To examine the influence of GM corn feeding on, the initiation and exacerbation of ovalbumin (OVA)-induced allergic asthma, we injected female BALB/c mice on days 0 and 21 with OVA intraperitoneally and nebulized them with OVA on days 28 and 29 to initiate disease and then allowed mice to recover until they were re-exposed to OVA for the induction of a disease exacerbation. We fed mice pellets containing 33% Bt (MON810)- or isogenic-corn vs. normal mouse food containing no corn for 4 weeks prior to inducing disease or inducing disease exacerbation. To evaluate the effects of the Bt-corn on OVA-induced disease, we measured lung and airway inflammation, mucus hypersecretion and OVA-specific antibodies. We observed that Bt-corn feeding had no effect on OVA-induced allergic disease or exacerbations indicating that Bt-corn using this protocol has no effect on the propensity for another allergen to initiate allergic disease or induce disease exacerbations in mice.

